# Medical students attitudes toward and intention to work with the underserved: a systematic review and meta-analysis

**DOI:** 10.1186/s12909-021-02517-x

**Published:** 2021-02-24

**Authors:** Edouard Leaune, Violette Rey-Cadilhac, Safwan Oufker, Stéphanie Grot, Roy Strowd, Gilles Rode, Sonia Crandall

**Affiliations:** 1 Faculté de Médecine Lyon-Est, Université de Lyon, Lyon, France; 2 Centre Hospitalier le Vinatier, 95 boulevard Pinel BP 300 39 -, 69 678 Bron cedex, France; 3 INSERM, U1028; CNRS, UMR5292, Lyon Neuroscience Research Center, F-69000 Lyon, France; 4 Centre de recherche, Institut Universitaire en Santé Mentale de Montréal, Montreal, Canada; 5 Wake Forrest School of Medicine, Winston-Salem, North Carolina USA

**Keywords:** Underserved, Attitudes, Medical students, Medical education, Community-based learning

## Abstract

**Background:**

Experts in the field of medical education emphasized the need for curricula that improve students’ attitudes toward the underserved. However, some studies have shown that medical education tends to worsen these attitudes in students. We aimed at systematically reviewing the literature assessing the change in medical students’ attitudes toward the underserved and intention to work with the underserved throughout medical education, the sociodemographic and educational factors associated with favorable medical student attitudes toward and/or intention to work with the underserved and the effectiveness of educational interventions to improve medical student attitudes toward and/or intention to work with the underserved.

**Method:**

We conducted a systematic review on MEDLINE, Scopus, and Web of Science databases. Three investigators independently conducted the electronic search. We assessed the change in medical students attitudes toward the underserved by computing a weighted mean effect size of studies reporting scores from validated scales. The research team performed a meta-analysis for the sociodemographic and educational factors associated with medical students attitudes toward and/or intention to work with the underserved.

**Results:**

Fifty-five articles met the inclusion criteria, including a total of 109,647 medical students. The average response rate was 73.2%. Most of the studies were performed in the USA (*n* = 45). We observed a significant decline of medical students attitudes toward the underserved throughout medical education, in both US and non-US studies. A moderate effect size was observed between the first and fourth years (d = 0.51). Higher favorable medical students attitudes toward or intention to work with the underserved were significantly associated with female gender, being from an underserved community or ethnic minority, exposure to the underserved during medical education and intent to practice in primary care. Regarding educational interventions, the effectiveness of experiential community-based learning and curricula dedicated to social accountability showed the most positive outcome.

**Conclusions:**

Medical students attitudes toward the underserved decline throughout medical education. Educational interventions dedicated to improving the attitudes or intentions of medical students show encouraging but mixed results. The generalizability of our results is impeded by the high number of studies from the global-North included in the review.

**Supplementary Information:**

The online version contains supplementary material available at 10.1186/s12909-021-02517-x.

## Background

The term “underserved” is used to define vulnerable and at-risk populations, especially individuals who are uninsured, poor, from racial and ethnic minorities, homeless, newly immigrated, socially isolated, or poorly educated [1]. Many studies have demonstrated that underserved populations experience health disparities, including increased premature mortality [2], poorer physical [3] and mental health status [4], and diminished access to the healthcare system and health prevention programs [5]. Currently, methods of healthcare delivery in many countries limit the ability to provide optimal care to these populations. To reduce health disparities, programs are needed that cultivate a strong sense of social accountability and improve medical students’ competency in providing care for the underserved [6–8]. Smitherman et al. [9] recently advocated for a new framework for medical schools – beyond the traditional tripartite mission of education, research, and clinical care – to include a fourth mission, social accountability. This new framework implies that medical schools have the mission to cultivate social accountability in medical students in order to participate in the improvement of health conditions of the underserved.

As stated by Boelen and Woollard in 2009 [10], socially accountable actions of medical schools must be grounded in the identification of societal needs and directed to the usability of professionals to fulfill those needs. According to the REVOLUTIONS framework developed by Ventres and Dharamsi [11], socially accountable medical schools must promote organizational cultures aimed at addressing social determinants of health to encourage the development of socially responsive physicians. Socially accountable medical students should know about social determinants of health, understand methods for delivering care to the underserved, participate in programs to broaden care delivery, and show favorable attitudes toward this population. Two concepts found in the literature to assess how medical students perceive their responsibility toward underserved populations and how they interact with them are medical students’ attitudes toward the underserved (MS-ATU) [12, 13] and future intention to work with the underserved (MS-IWU) [14]. MS-ATU refers to the perceptions that medical students have of underserved populations and their health conditions and behaviors [12, 13]. MS-IWU defines the willingness displayed by medical students to work in socially deprived areas or with underserved populations after graduating [14]. Several tools showed good validity to evaluate how medical students feel responsible for the care of underserved populations and how they conceive social issues in medicine [12, 15–18].

However, since the pioneering work by Eron in 1955 [19], studies continue to report that progressing through medical education may negatively impact attitudes toward caring for underserved patients [12, 13]. In 1993, Crandall, Volk and Loemker asked the following critical question for both medical educators and healthcare policies makers: « Are we training socially responsible physicians? » [12]. Moreover, the factors associated with favorable MS-ATU, which may limit the negative impact of medical education on students’ attitudes and intention, are poorly understood. More than 25 years later, the question not only remains but is increasingly important as social media, telehealth, and technology are changing healthcare delivery and training. However, no previous systematic review addressed the issue of MS-ATU and/or MS-IWU, regarding their change throughout medical education and the factors associated with favorable MS-ATU and MS-IWU.

During the past years, medical schools implemented selection strategies or educational interventions (curricula on social accountability, community-based learning, lectures…) dedicated to social accountability, with the goal to train competent physicians showing more favorable attitudes toward and intention to work with the underserved [20, 21]. However, no systematic review has yet summarized the evidence on the effectiveness of such strategies for improving MS-ATU and/or MS-IWU.

Our systematic review and meta-analysis aimed to examine the three following questions:

1. How do MS-ATU and MS-IWU change throughout medical education?
2. How do sociodemographic and educational factors predict MS-ATU and MS-IWU?
3. What types of interventions used by medical schools show the best effectiveness to improve MS-ATU and MS-IWU?

## Method

### Article selection

The systematic review protocol was based on the Preferred Reporting Items for Systematic Reviews and Meta-analyses (PRISMA) protocol [22] and registered on the PROSPERO database (registration number CRD42019120628), in March 2019. The electronic search was conducted on the following databases: Medline, Scopus, and Web of Science. The following search terms were grouped by subject as follows: a) context of medical education; b) outcomes: change in MS-ATU and/or MS-IWU, predictive factors for MS-ATU and/or MS-IWU, effectiveness of educational interventions; c) underserved population. The search algorithm is given in Additional file 1: appendix. Search terms were connected using the Boolean Operators ‘AND’ and ‘OR’ to capture all relevant article suggestions. To ensure the exhaustiveness of the search and the relevance of the algorithm, a pilot electronic research was performed. The results of the pilot search were reviewed and the search terms and algorithm were further refined.

The search was conducted independently by three investigators (EL, VRC, and SO) using the same criteria and search procedures between January and May 2020. The electronic search was supplemented by a manual search of references in relevant articles and by snowballing of full articles retrieved to maximize identification of relevant literature. Three selection filters were used: titles, abstracts, and a comprehensive full-text reading of each selected article thereafter. At each stage, the authors independently screened all the selected articles, identified and selected those for which available data (title for the first stage, abstract for the second and then full-text) were consistent with the inclusion criteria of the review. The three authors compared their results at each stage of the selection process and agreed upon the articles to be retained. When the three investigators disagreed about the inclusion of an article, we reached agreement through consensus among all authors.

### Inclusion criteria

We included articles that (1) were published in peer-reviewed journals in English, (2) were published until 31 December 2019, (3) reported the change in MS-ATU and/or MS-IWU throughout medical education, (4) assessed factors associated with MS-ATU and/or MS-IWU, or (5) evaluated effectiveness of educational interventions to improve MS-ATU and/or MS-IWU. Cross-sectional, cohort, and interventional studies (including randomized trials) were eligible for inclusion. We excluded articles reporting non-original data and qualitative studies. Two authors assessed the quality of studies included through a rating scheme modified from the Oxford Centre for Evidence-based Medicine [23]. For interventional studies, we also assessed study quality using the Medical Education Research Study Quality Instrument (MERSQI), developed to assess the methodologic quality of quantitative medical education research [24]. The MERSQI shows good interrater reliability (interrater reliability comprised between 0.77 and 0.95) [24]. The instrument uses 10 criteria: study design, number of institutions included, response rate, data type, internal structure, content validity, criterion validity, appropriateness of data analysis, complexity of analysis, and outcome level. These criteria form six domains, each with a maximum score of 3 and a minimum of 0 or 1, that sum to produce a total score from 5 to 18.

### Data analysis

#### Systematic review

The first three authors independently extracted quantitative data for each outcome: change in MS-ATU and MS-IWU; mediating factors associated with favorable MS-ATU; and effectiveness of educational strategies dedicated to improving MS-ATU. We reviewed the extracted results, grouped them into main categories, and summarized the evidence for each category. We then discussed all discrepancies and reached consensus on the final summary. When necessary, we contacted original authors of included studies to give detailed information on incomplete data (e.g.*,* sample size, response rates, results of the study). In the literature, several validated scales are used to evaluate MS-ATU, such as the Medical Student Attitudes Towards the Underserved (MSATU) [12, 13], the Attitudes Toward Issues in Medicine (ATSIM) [15, 16] and the Attitudes Toward Poverty (ATP) [17, 18]. MS-IWU is generally assessed through non-validated questionnaire asking medical students if they intent or not to work with the underserved [14].

#### Change in MS-ATU

We assessed the change in MS-ATU by computing a weighted mean effect size of studies reporting results from the ATSIM, MSATU and ATP scales. For each study, effect size was calculated as the standardized mean difference (Cohen’s d) between the first year of medical school and the later years. A positive effect size reflects lower scores (ATSIM and MSATU) or a higher score (ATP), respectively. Conventionally, Cohen’s d of 0.20, 0.50, and 0.80 are considered as small, moderate, and large effects, respectively [25].

#### Meta-analysis

When results were given in odds ratios (OR) and had homogeneous outcomes, we analyzed data using Comprehensive Meta-analysis Software (V2.0, Biostat) [26]. We made the choice of using ORs according to recent recommendations regarding the summary analysis of dichotomous outcomes in meta-analysis [27], to fulfill the four following criteria: 1) consistency across studies, 2) mathematic properties to perform a meta-analysis, 3) ease of interpretation for the readers and 4) availability of data as study-level summaries. Indeed, as the great majority of the included articles used ORs to report their results as study-level summaries regarding the factors associated with favorable MS-ATU, the used of ORs was indicated to perform the meta-analysis. We measured pooled ORs using a random-effect model, to consider between-study variability and thus provide more precisely estimated summary ORs [28]. For each study, we converted ORs into the log OR. The weighted sum of the log ORs was measured and then reconverted into ORs. We quantified heterogeneity among studies with the Q statistic and the I2. The Q statistic determines whether observed variations in OR are caused by a between-study true difference, not within-study sampling error. A significant Q-value reflects a true variation of OR between studies. I2 is the proportion of inconsistency between studies’ results attributable to heterogeneity. I2 values of 25, 50, and 75% reflect a small, moderate, or high degree of heterogeneity, respectively [29]. We assessed publication bias with the Egger’s regression intercept [30].

## Results

### Included studies

**Fig. 1:**
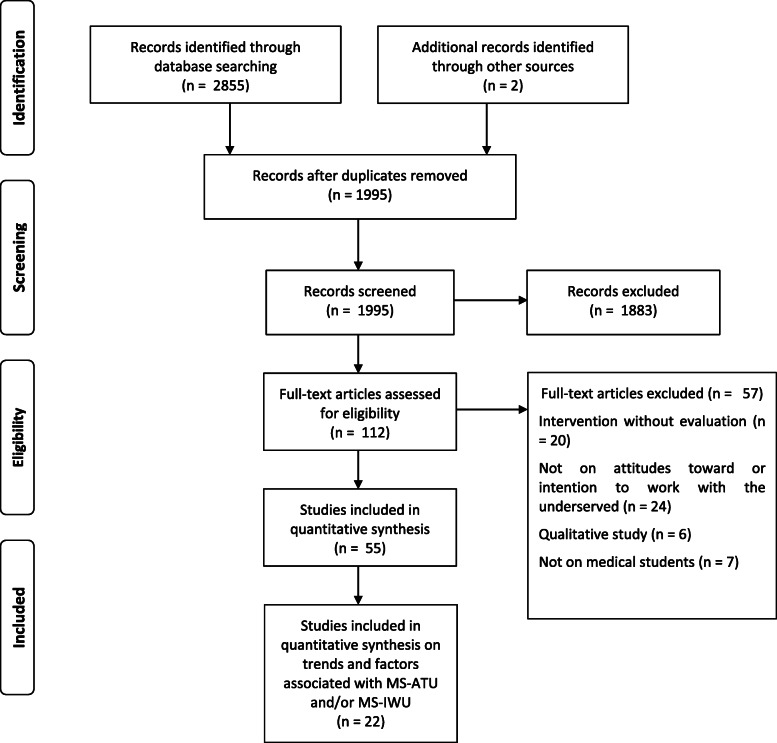
Flowchart diagram of study selection according to the PRISMA guidelines

After removing duplicate citations, 1781 articles were identified. After selection on title and abstract, followed by full-text reading, 55 articles met our inclusion criteria [12–14, 16, 18, 21, 22, 31–77, 78]. Of these, twelve studies measuring the trends in MS-ATU [12, 13, 18, 32, 33, 34, 43, 45, 47, 51, 54, 63] and 10 focusing on factors associated with MS-ATU [42, 50, 52, 56, 59, 64, 66–68, 71] were included in the quantitative analysis (Fig. 1).

Studies were published between 1980 and 2019. The 55 studies included 109,647 medical students, 101,327 of whom were U.S. medical students (92.4%). Most studies were performed in the USA (*n* = 45) or Australia (*n* = 6). The average response rate was 73.2%. The quality of studies was heterogeneous. The characteristics of included studies are displayed in Table 1.

**Table 1:** Characteristics of studies included in the systematic review and meta-analysis

Source	Country	Design	Sample	Response rate	Outcome	Quality^**a**^
**Maisiak et al. 1980** [31]	US	Non-controlled trial	145	88%	Effectiveness of an educational intervention	2
**Dornbush et al. 1985** [16]	US	Cross-sectional	144	72%	Change in MS-ATU	4
**Ewan et al. 1987** [32]	AU	Cross-sectional	156	71.2%	Change in MS-ATU	4
**Ewan et al. 1988** [33]	AU	Cohort	63	68%	Change in MS-ATU	2
**Dornbush et al. 1991** [34]	US	Cross-sectional	71	38%	Change in MS-ATU	4
**Crandall et al. 1993** [12]	US	Cross-sectional	220	80.9%	Change in MS-ATU	4
**Tippets & Westphelling**^**,**^ **1993** [35]	US	Cross-sectional	560	NA	Mediating factors	4
**Campos-Outcalt et al. 1997** [36]	US	Case-control	193	68.4%	Effectiveness of an educational intervention	3
**Crandall et al. 1997** [13]	US	Cohort	495	80%	Change in MS-ATU	2
**O’Toole et al. 1999** [37]	US	Cohort	160	68.5%	Effectiveness of an educational intervention	2
**Weitzmann et al. 2000** [38]	US	Cross-sectional	141	71%	Change in MS-IWU	4
**Markham et al. 2001** [39]	US	Non-controlled trial	90	90.9%	Effectiveness of an educational intervention	3
**Weissman et al. 2001** [40]	US	Cross-sectional	2626	65.3%	Mediating factors	4
**Godkin et al. 2003** [41]	US	Non-controlled trial	146	83.4%	Effectiveness of an educational intervention	3
**Tavernier et al. 2003** [42]	US	Cross-sectional	775	26.3%	Mediating factors	4
**Schwartz & Loten, 2004** [43]	NZ, US	Cross-sectional	1015	NA	Change in MS-ATU	4
**Wilson et al. 2004** [44]	US	Cross-sectional	784	57%	Change in MS-ATU	4
**Woloschuk et al. 2004** [45]	CA	Cohort	198	52.5%	Change in MS-ATU	2
**Ko et al. 2005** [20]	US	Controlled trial	1088	93.6%	Effectiveness of an educational intervention	2
**Cox et al. 2006** [46]	US	Randomized trial	100	93%	Effectiveness of an educational intervention	1
**Godkin et al. 2006** [47]	US	Controlled trial	196	98.0%	Change in MS-ATU + Effectiveness of an educational intervention	2
**Buchanan et al. 2007** [48]	US	Non-controlled trial	25	100%	Effectiveness of an educational intervention	3
**Crandall et al. 2007** [49]	US	Cohort	110	71%	Change in MS-ATU + Effectiveness of an educational intervention	2
**Ko et al. 2007** [50]	US	Case-control	1071	100%	Effectiveness of an educational intervention + Mediating factors	3
**Crandall et al. 2008** [51]	US	Cohort	110	100%	Change in MS-ATU	2
**Dyrbye et al. 2010** [52]	US	Cross-sectional	2682	61%	Mediating factors	4
**Huang & Malinow, 2010** [53]	US	Non-controlled trial	46	100%	Effectiveness of an educational intervention	3
**Wayne et al. 2011** [54]	US	Cohort	313	59%	Change in MS-ATU	2
**Scheu et al. 2012** [55]	US	Non-controlled trial	274	75%	Effectiveness of an educational intervention	3
**Boscardin et al. 2014** [56]	US	Cohort	7631	58.8%	Mediating factors	2
**Caulfield et al. 2014** [57]	US	Cross-sectional	13,867	74.2%	Mediating factors	4
**Smith et al. 2014** [58]	US	Non-controlled trial	914	97.9%	Effectiveness of an educational intervention	3
**Borracci et al. 2015** [59]	AR	Cross-sectional	354	88.5%	Mediating factors	4
**Cox et al. 2015** [60]	US	Randomized controlled trial	137	88%	Effectiveness of an educational intervention	1
**Jilani et al. 2015** [18]	US	Cross-sectional	297	67%	Change in MS-ATU	4
**Girotti et al. 2015** [61]	US	Case-control	297	36%	Effectiveness of an educational intervention	3
**Larkins et al. 2015** [62]	AU, BE, CA, PH, ZA, SD	Case-control	944	88.9%	Effectiveness of selection strategies	3
**Stephens et al. 2015** [63]	US	Cross-sectional	170	35%	Change in MS-ATU	4
**Laraque Arena et al. 2016** [64]	US	Cross-sectional	1223	57%	Mediating factors	4
**Leung et al. 2016** [65]	US	Non-controlled trial	48	72%	Effectiveness of an educational intervention	3
**Gatell et al. 2017** [66]	US	Cross-sectional	393	66%	Mediating factors	4
**Griffin et al. 2017** [14]	AU	Cohort	351	94.6%	Mediating factors	2
**O’Connell et al. 2017** [67]	US	Cohort	564	70.4%	Change in MS-IWU + mediating factors	2
**Puddey et al. 2017** [68]	AU	Cross-sectional	2829	89.8%	Mediating factors	4
**Tran et al. 2017** [69]	US	Controlled trial	128	NA	Effectiveness of an educational intervention	2
**Briggs et al. 2018** [70]	US	Controlled trial	42	88%	Effectiveness of an educational intervention	2
**Garcia et al. 2018** [71]	US	Cross-sectional	40,846	100%	Change in MS-IWU + Mediating factors	4
**Kost et al. 2018** [72]	US	Case-control	158	77%	Effectiveness of an educational intervention	3
**Larkins et al. 2018** [21]	AU, BE, CA, PH, ZA, SD	Controlled trial	3346	76.2%	Effectiveness of an educational intervention	2
**Wolley et al. 2018** [73]	PH	Case-control	492	45.5%	Effectiveness of selection strategies	3
**Burkhardt et al. 2019** [74]	US	Cross-sectional	17,067	91%	Mediating factors	4
**Godfrey et al. 2019** [75]	US	Non-controlled trial	59	84%	Effectiveness of an educational intervention	3
**Heller et al. 2019** [76]	ZA	Non-controlled trial	52	NA	Effectiveness of an educational intervention	3
**Jacobs et al. 2019** [77]	US	Non-controlled trial	11	50%	Effectiveness of an educational intervention	3
**Phelan et al. 2019** [78]	US	Cohort	3756	64.5%	Change in MS-IWU	2

^a^ The quality of included studies was measured using the rating scheme modified from the Oxford Centre for Evidence-based Medicine^23^: the score is based on the design of the study (1 = randomized trial; 2 = controlled trial without randomization; 3 = case-control study or retrospective cohort study; 4 = cross-sectional study or case series)

Abbreviations: *MS-ATU* Medical students attitudes toward the underserved; *MS-IWU* Medical students intention to work with the underserved; *AR* Argentina; *AU* Australia; *BE* Belgium; *CA* Canada; *NZ* New Zealand; *PH* Philippines; *ZA* South Africa; *SD* = Sudan; *US* United States of America

### Change in MS-ATU and MS-IWU

#### Change in MS-ATU (Table 2)

Thirteen studies [12, 13, 18, 32–34, 43–45, 47, 51, 54, 63] measured change in MS-ATU using three different scales. One study did not use any validated scale [44]. All but one study [32] reported decreasing MS-ATU throughout medical education. Eleven [12, 13, 16, 18, 32–34, 45, 47, 51, 63] studies assessed the significance of the change; it was significant in all but 2 [16, 32].

**Table 2:** Change in medical students attitudes toward the underserved (MS-ATU) throughout medical education

Author	Country	Scale	1st year	2nd year	3d year	4th year	5th year	Mean difference (%)	Significance
**Dornbush et al. 1985** [16]	US	ATSIM^a^	168.11	–	165.85	–	–	**−2.26 (1.3%)**	*NS*
**Ewan, 1987** [32]	Australia	ATSIM	175.9	–	–	179.4	–	**+ 3.5 (1.9%)**	*NS*
**Ewan, 1988** [33]	Australia	ATSIM	–	–	–	176	167	**−9.0 (5.1%)**	*p < 0.001*
**Dornbush et al. 1991** [34]	US	ATSIM	213.81		195.15	–	–	**−18.66 (8.7%)**	*p = 0.037*
**Crandall et al. 1993** [12]	US	MSATU^a^	66.3	–	–	60.8	–	**−5.5 (8.3%)**	*p < 0.001*
**Crandall et al. 1997** [13] **(women)**	US	MSATU	54.1	48.4	47.6	43.5	–	**−10.6 (22.2%)**	*p < 0.001*
**Crandall et al. 1997** [13] **(men)**	US	MSATU	48.6	44.4	44.7	40.9	–	**−7.7 (15.8%)**	*p < 0.001*
**Schwartz & Loten, 2004** [43]	New Zealand	ATSIM	13.46	13.61	12.9	–	–	**− 0.56 (4.2%)**	*NA*
**Schwartz & Loten, 2004** [43]	Hawaii	ATSIM	14.75	14.54	14.21	14.31	–	**−0.44 (2.9%)**	*NA*
**Schwartz & Loten, 2004** [43]	Australia	ATSIM	13.21	–	13	–	–	**−0.21 (1.6%)**	*NA*
**Wolloschuk et al. 2004** [45]	Canada	ATSIM	187.2	182.01	–	176.05	–	**−11.15 (5.9%)**	*p < 0.001*
**Godkin et al. 2006** [47]	US	MSATU	3.98	–	–	3.81	–	**−0.17 (4.3%)**	*p < 0.05*
**Crandall et al. 2008** [51]	US	MSATU	52.8	51.4	–	46.6	–	**−6.2 (11.7%)**	*p < 0.01*
**Wayne et al. 2011** [54]	US	MSATU	54.9	–	–	50.4	–	**−4.5 (8.2%)**	*NA*
**Jilani et al. 2015** [18]	US	ATP^b^	48.15	–	–	51.69	–	**+ 3.54 (7.4%)**	*p = 0.016*
**Stephens et al. 2015** [63]	US	MSATU	46.02	–	–	41.7	–	**−4.32 (9.4%)**	*p < 0.01*

*MSATU* Medical Student Attitude Toward the Underserved scale; *ATSIM* Attitudes Toward Social Issues in Medicine scale; *ATP* Attitudes Toward Poverty*

^a^ Higher score in ATSIM and MSATU indicate a more favorable attitude toward the underserved^15,16^

^b^ Higher score in ATP indicate a less favorable attitude toward the underserved^17,18^

Twelve studies were included in the measure of effect sizes [12, 13, 18, 32–34, 43, 45, 47, 51, 54, 63]. The heterogeneity between studies was high (I^2^ = 72.44; Q(8) = 29.02; *p* < 0.001). We found a small effect size between the first and second years (d = 0.29) and between the first and third years (d = 0.34). A moderate effect size was observed between the first and fourth years (d = 0.51). Decline in MS-ATU was reported to be greater in men than women [12, 13, 34, 54], between years 1 and 2 and then between years 3 and 4 of medical education [13]. Students from underserved backgrounds and those interested in primary care showed less decline in MS-ATU compared to their counterparts [54]. Crandall et al. [51] showed that the decline throughout education was more pronounced in medical students than pharmacy students. The decline in MS-ATU was observed both in older and more recently published studies [16, 32–34, 54, 63]. Three studies found that the decline in MS-ATU was observed not only in the US but also in other countries (Australia, Canada and New-Zealand) [33, 43, 45], while Ewan [32] reported a non-significant increase in MS-ATU in Australia.

#### Change in MS-IWU

Four studies [38, 67, 71, 78] evaluated the change in MS-IWU in the US with conflicting results. Garcia et al. [71] and Phelan et al. [78] reported an increase in MS-IWU between matriculation and graduation (23 to 28% and 22.5 to 23.1%, respectively) and O’Connel et al. [67] an increase between the third and fourth year (32 to 34.3%). Weitzmann et al. [38] reported a decrease in MS-IWU between the beginning and third year of pediatric residency. None of the four studies assessed the significance of change in MS-IWU. Phelan et al. [78] found a significant association between decrease in MS-IWU and more negative fourth-year explicit racial attitudes.

### Factors associated with favorable MS-ATU or MS-IWU

A total of 16 articles [14, 35, 40, 42, 50, 52, 57, 59, 64, 66, 67, 68, 71, 74, 78] assessed the role of mediating factors associated with MS-ATU or MS-IWU, identifying 13 main putative mediating factors. Ten studies were included in the meta-analysis [﻿49﻿, 42, 50, 52, 56, 59, 64, 66, 67, 68, 71], 4 were excluded because they did not report their results in ORs [35, 40, 57] and 2 because they assessed a risk factor that was not evaluated in other studies [74, 78].

The heterogeneity between studies was high (I^2^ = 78.1%; Q(7) = 32.0; *p* < 0.001). Pooled ORs showed greater attitudes and intentions for female gender, being from an underserved community, being from ethnic minority, exposure to the underserved during medical education and intent to practice in primary care (Table 3). The association was not significant for age, religiosity, or educational debt.

**Table 3:** Pooled odd ratios for sociodemographic and educational factors associated with favorable medical students attitudes toward (MS-ATU) or intention to work with the underserved (MS-IWU)

Factor	n	Pooled OR (95% CI)	95% CI	z-value	***p***
**Gender (female)**	7	1.47	1.18–1.81	3.52	< 0.001
**Students from underserved communities**	6	2.20	1.51–3.21	4.12	< 0.001
**Students from ethnic minorities**	5	2.06	1.64–2.60	6.15	< 0.001
**Age**	5	1.05	0.95–1.15	0.96	0.34
**Intention to work in primary care**	5	1.97	1.27–3.05	3.05	0.002
**Exposure to the underserved during medical education**	4	1.47	1.26–1.71	4.86	< 0.001
**Religiosity**	3	0.93	0.65–1.35	−0.36	0.72
**Educational debt**	3	0.93	0.73–1.19	−0.60	0.55

*MS-ATU* Medical students attitudes toward the underserved; *MS-IWU* Medical students intention to work with the underserved

Two studies evaluated the role of burnout on MS-ATU, with conflicting results. Dyrbye et al. [52] found a significant association between greater burnout and unfavorable MS-ATU (OR = 1.65; 95% CI 1.35–2.01); whereas Gatell et al. [66] did not (OR = 1.3; 95% CI 0.7–2.6). Two studies found a significant association between sense of calling and MS-IWU [67, 68]. Burkhardt et al. [74] reported greater MS-IWU in residents who plan a career in emergency medicine at graduation (OR = 1.71; 95% CI 1.57–1.87). Phelan et et al. [78] found no significant association between the experience of microaggressions attributed to race/ethnicity and MS-IWU (OR = 1.24; *p* = 0.24). Studies that were not included in the meta-analysis because they did not report their results in ORs also found more favorable MS-ATU in females [14, 35, 40], medical students from underserved communities [35, 40], or ethnic minorities [35, 40] and those who intent to practice in primary care [35]. Griffin et al. [14] reported a greater MS-IWU in students with middle socio-economic status compared to those with low or high socio-economic status (*p* < 0.05). Caulfield et al. [57] found an association between tolerance of ambiguity and favorable MS-ATU (d = 0.29).

### Effectiveness of educational interventions and selection strategies (Table 4)

#### Effectiveness of educational interventions

A total of 24 articles assessed the effectiveness of educational interventions [20, 21, 31, 36, 37, 39, 41, 46–48, 50, 51, 53, 55, 58, 60, 61, 65, 69, 70, 72, 75–77]. Twenty were US studies. Thirteen were controlled-group studies [20, 21, 36, 37, 46, 47, 49, 50, 60, 61, 59, 70, 72], among which two were randomized [46, 60]. Five main types of educational interventions were evaluated: global curriculum dedicated to social accountability [20, 21, 36, 46, 47, 48, 50, 51, 53, 60, 61, 72, 77]; experiential community-based learning [37, 41, 70]; volunteering in student-run free clinics [55, 58, 69, 76], traditional didactic learning^,,^ [31, 39, 65] and online curriculum [75]. Experiential community-based learning and global curricula showed the greatest positive impact on MS-ATU, whereas volunteering in student-run free clinics and traditional learning showed inconsistent results.

**Table 4:** Characteristics and results of studies evaluating the effectiveness of interventions dedicated to improving medical students attitudes toward (MS-ATU) or intention to work with the underserved (MS-IWU)

Source	Medical school	Educational program or selection strategies	Duration	Outcome	Results	Quality^**a**^
**Global curriculum**						
Campos-Outcalt et al. 1997 [36]	University of Arizona	“Commitment to Underserved People” Program • Visits to agencies providing services to the underserved (Y1) • Volunteer projects and community health externship (Y1–2) • Assistance in selecting site for Y3 clerkships • Primary-care preceptorships among underserved populations (Y4) • Discussions and annual retreat for faculty and students (Y1–4)	4y	Practice in underserved area	Improved**	11.5
Ko et al. 2005 [20]	University of California, Charles R. Drew University	UCLA/Drew Medical Education Program • Clinical placement in underserved areas • Longitudinal primary care rotation • Research project in an underserved area • Didactics on minority and multicultural health • Health disparities thesis	4y	MS-IWU	Improved**	9.5
Cox et al. 2006 [46]	University of Wisconsin School of Medicine	“Caring for the Underserved” Curriculum • Faculty-led and web-based curriculum for third-year medical students during the pediatric clerkship • Case-based videos & interactive problem-based learning • Development of a screening tool for recognizing underserved patients (“I CARE”) • Independent clinical project on the situation of an underserved family met during a clinic visit	6w	MS-ATU	Improved*	10.5
Godkin et al. 2006 [47]	University of Massachusetts Medical School	“Longitudinal Pathway on Serving Multicultural and Underserved Populations” • Family curriculum (Y1): visits to an underserved family • Longitudinal 2-year preceptorship (Y1–2): placement at a site serving underserved populations • Summer immersion: 6-week immersion in a developing country (at the end of Y1) • Domestic community service project family medicine clerkship (Y3): serving in or organizing a community service project for the underserved • Electives (Y3–4): placement in a community or rural health center and/or in a developing country • Seminar series	4y	MS-ATU	Unchanged^b^	11.5
Buchanan et al. 2007 [48]	Rush University, Chicago	Primary care internal residency program • Series of 8 lectures on homeless’ conditions • Journaling, discussions with homeless individuals • Tours of community programs serving homeless people • Intensive clinic sessions in homeless shelters and visits to service providers	2w	MS-ATU	Improved***	11.5
Ko et al. 2007 [50]	University of California, Charles R. Drew University	UCLA/Drew Medical Education Program • Clinical placement in underserved areas • Longitudinal primary care rotation • Research project in an underserved area • Didactics on minority and multicultural health • Health disparities thesis	2y	Practice in underserved area	Improved*	12.5
Crandall et al. 2007 [51]	Wake Forrest University School of Medicine	• Preclinical problem-based learning curriculum	4y	MS-ATU	Unchanged^b^	11.5
Huang & Malinow, 2010 [53]	Baylor College of Medicine	“Longitudinal Ambulatory Care Experience Underserved Pathway” • Half-day seminar/journal clubs on underserved care topics • Clinical preceptorship: visits to a preceptor practicing in an underserved area • Community visits: group visits to organizations caring for the underserved • Year-long group project: development of a community-based intervention for the underserved • Health care funding assignment: assistance to a patient applying for health care funding	1y	MS-ATU	Improved*	7
Cox et al. 2015 [60]	University of Wisconsin School of Medicine	“Caring for the Underserved” Curriculum • Faculty-led and web-based curriculum for third-year medical students during the pediatric clerkship • Case-based videos & interactive problem-based learning • Development of a screening tool for recognizing underserved patients (“I CARE”) • Independent clinical project on the situation of an underserved family met during a clinic visit	6w	MS-ATU	Unchanged^c^	10
Girotti et al. 2015 [61]	University of Illinois College of Medicine	“Urban Medicine Program” • Seminar series (Y1–2): didactic learning followed by breakout sessions • Web-based learning curriculum (Y3–4): three modules covering cultural competency, leadership and communication skills • Longitudinal community project (Y3–4): team-based collaborative engagement with community agencies in underserved areas • Policy and advocacy forum (Y4): two-weeks didactic presentations and discussions	4y	MS-ATU	Improved*	15
Larkins et al. 2018 [21]	James Cook University, Townsville (AU) Ghent University (BE) Walter Sisulu University (ZA) Gezira University (SD) Ateneo de Zamboanga School of Medicine (PH) Northern Ontario School of Medicine (CA) Flinders University School of Medicine (AU) University of the Philippines School of Health Sciences (PH)	Training for Health Equity Network (THEnet) • Medical schools with a social accountability mandate • Didactics on social determinant of health and health needs of underserved people • Clinical placements in underserved areas	–	MS-IWU	Unchanged^b^	10
Kost et al. 2018 [72]	University of Washington School of Medicine	The Underserved Pathway Program • Online curriculum, • Preclinical underserved preceptorship • Electives on underserved topics • Mentorship with a physician with experience caring for underservedpopulations, • Service learning, • Scholarly project with an underserved focus, • Clinical clerkships at underserve sites	4y	MS-IWU	Improved*	11
Jacobs et al. 2019 [77]	Saint Louis University	The Longitudinal Underserved Community Curriculum (LUCC) • 12 monthly day-long community health workshops • Monthly community-focused 1-h seminars • Practice in a diverse urban federally qualified health center	3y	MS-ATU	Improved^b^	10
**Experiential learning**						
O’Toole et al. 1999 [37]	University of Pittsburgh	“Program for Health Care to Underserved Population” • Service-learning program at a health care clinic for homeless including patient admission, physical examination, health education talks, treatment	4y	MS-IWU	Improved***	8.5
Godkin et al. 2003 [41]	University of Massachusetts Medical School	• International electives	4-8w	MS-ATU	Improved***	8.5
Briggs et al. 2018 [70]	Geisel School of Medicine at Dartmouth	“Beyond the Book” Program • Classroom didactics (lectures, panelist and journal article discussions, workshops) • Community-based learning: partnership with underserved individuals, meetings in nonprofit organizations serving the underserved	8 m	MS-ATU	Improved***	11
**Volunteering in SRFC**						
Sheu et al. 2012 [55]	School of Medicine, University of California	• Classroom didactics • Volunteering in free clinics: performing histories and physicals, providing patient education	1y	MS-ATU	Unchanged^b^	10.5
Smith et al. 2014 [58]	University of California San Diego	Student-run Free Clinic Project • Half-day clinical sessions • Two-hours didactic sessions • Structured oral and written reflection activities • Clinical sessions including managing all functions of the clinic	–	MS-ATU	Improved***	7.5
Tran et al. 2017 [69]	University of Central Florida College of Medicine	• Volunteering at the KNIGHTS (Keeping Neighbors In Good Health Through Service) Clinic	–	MS-ATU and MS-IWU	Unchanged^b^	7.5
Heller et al. 2019 [76]	University of Cape Town School of Medicine	• Volunteering at the SHAWCO (Students’ Health and Welfare Centers Organisation)	–	MS-IWU	Unchanged^b^	11.5
**Traditional learning**						
Maisiak et al. 1980 [31]	University of Alabama in Birmingham	• Lectures on social determinants of health	10w	MS-ATU	Unchanged^b^	12
Markham et al. 2001 [39]	Jefferson Medical College, Thomas Jefferson University	• Seminar series (Y3): one-hour sessions of articles discussions about health care environment	4 h	MS-ATU	Improved^b^	7.5
Leung et al. 2016 [65]	Warren Alpert Medical School of Brown Uni-versity	• Seminars on topics related to the health of underserved population and collaboration on a presentation or workshop	1y	MS-ATU	Unchanged^b^	8
**Online curriculum**						
Godfrey et al. 2019 [75]	Columbia Vagelos of Physicians and Surgeon	• Six modules incorporating mobile-optimized media content: health systems, social determinants of health, race and health, injury and violence, substance use and harm reduction, alternative health systems and current health policy	5w	MS-IWU	Improved^c^	8.5
**Selection strategies**						**11.0**
Larkins et al. 2015 [62]	James Cook University, Townsville (AU) Ghent University (BE) Walter Sisulu University (ZA) Gezira University (SD) Ateneo de Zamboanga School of Medicine (PH)	• Selection on academic merit (AU, BE, ZA, SD, PH) • Interview by panel including members from underserved communities (AU, PH, ZA) • Quota system for students from underserved communities (AU, PH, SD, ZA) • Marketing strategy to attract socially minded students (BE)	–	MS-IWU	Unchanged^c^	10
Wooley et al. 2018 [73]	University of the Philippines School of Health Sciences (PH) Ateneo de Zamboanga School of Medicine (PH)	• Selection on academic merit • Interview • Quota-system for students from underserved communities	4y	Practice in underserved area	Improved***	12

^a^ Measured using the *Medical Education Research Study Quality Instrument* (MERSQI)^24^: MERSQUI score range from 5 to 18

* p < 0.05

** p < 0.001

*** p < 0.001

^b^ Not significant

^c^ No measure of significance

Abbreviations: *MS-ATU* Medical students attitudes toward the underserved; *MS-IWU* Medical students intention to work with the underserved; *SRFC Student-run free clinic; AU* Australia, *BE* Belgium; *CA* Canada; *PH* Philippines; *SU* Sudan; *ZA* = South Africa

#### Effectiveness of selection strategies

Two studies evaluated the impact of selection strategies at entry in medical schools on MS-IWU [62, 73] and showed inconsistent results. Both were non-US studies (one from Australia, Belgium, Canada, Philippines, South African and Sudan and one from Philippines). Larkins et al. [62] found no significant impact on MS-IWU, while Wooley et al. [73] found a significant increase in MS-IWU in two Philippines medical schools.

### Publication bias

There was no evidence of publication bias for change in MS-ATU (r = 1.76; *p* = 0.34) or factors associated with favorable MS-ATU (r = − 0.97; *p* = 0.44).

## Discussion

We performed the first systematic review and meta-analysis on medical student attitudes toward the underserved throughout medical education. First, our systematic review demonstrated that MS-ATU significantly declines throughout medical education. Second, we found that factors associated with more favorable attitudes toward the underserved were sociodemographic characteristics, such as gender and social origin, not related to medical education. Third, community-based interventions were the only clear educational strategies that significantly improved MS-ATU. Experiential community-based learning and curricula dedicated to social accountability tended to show the highest levels of effectiveness in improving attitudes and intentions.

### Decline of MS-ATU

While speculative, our data suggest that declines in MS-ATU may represent an erosion of social responsibility that occurs during medical training. Future studies could seek to improve knowledge and attitudes toward social determinants of health and incorporate efforts to understand the social contract of physicians. Since students from other disciplines tend to display more favorable attitudes toward the underserved, interprofessional learning may also be an effective way to train socially accountable medical students. Moreover, no studies assessed the impact of interventions that include patients as educators in social accountability, which could be another method to train students in this topic.

We demonstrated that attitudes toward the underserved in medical students were not correlated to age of participants. We can thus assume that the maturation effect, i.e. change in their way of thinking as a result of getting older, may not explain the observed decline in MS-ATU. What specifically influences the decline remains unclear. Possible factors include the hidden curriculum, the intensity of the educational program, faculty and staff role models, or others. The hidden curriculum was originally defined in the field of medical education as the « set of influences that function at the level of organizational structure and culture » [79]. According to a recent scoping review, the hidden curriculum may be responsible for many negative outcomes in medical education, from erosion of idealism to increase in cynicism and bias in medical students [80]. Interestingly, Crandall et al. [51] showed that attitudes toward the underserved were maintained in pharmacy students. This is consistent with the findings of Parlow and Rothman [15], who reported more favorable attitudes over time toward societal responsibility in social work and nursing students. These data raise questions about the impact of medical school curricula on training socially aware clinicians.

As students progress through training, they experience increasing intensity in demands and time requirements which contribute to emotional exhaustion and burnout. The significant decline in empathy previously demonstrated throughout medical education [81] may also be a putative explanation for our findings, although no studies assessed associations between empathy and MS-ATU. While faculty and staff role models are known to play a key role in medical students’ attitudes [82], Wilson et al. [44] reported that physicians tend to show less favorable MS-ATU than students. We could thus hypothesize that negatives attitudes toward the underserved in senior physicians and medical educators may shape unfavorable MS-ATU through role model throughout medical education. These factors may also lead to a shift in focus away from their responsibility to society or underserved populations (if they do not interact with them daily) toward a more direct and immediate focus on the patient in front of them.

### Mediating factors

Sociodemographic characteristics, such as gender or social origin, were more strongly associated with positive attitudes toward the underserved than factors related to medical education. This result may represent an argument for selection strategies in medical schools to build a workforce of physicians willing to work with the underserved. However, the study of Larkins et al. [21] did not provide strong evidence that selection strategies influenced students’ maintaining a strong intention to work with the underserved. On the contrary, Wolley et al. [73] reported that selection strategy had a significant effect on the choice to work in an underserved area in the Philippines. Interestingly, interventions evaluated by Wolley et al. [73] used both a selection strategy and featured a curriculum dedicated to social accountability. However, ethical concerns have also been raised about selection strategy [83]. Students from underserved communities may indeed want to work outside underserved areas [14] and cannot be expected to limit their practice to such communities.

### Educational interventions

Studies failed to show significant differences between students who did and did not participate in programs teaching social accountability. Thus, it is challenging to measure effectiveness of these interventions in modifying medical students’ attitudes. According to our results, experiential learning may be one of the most effective ways to improve MS-ATU. Accordingly, a recent systematic review of Doobay-Persaud et al. [84] found that experiential learning significantly improved medical students’ knowledge of health inequities. However, although experiential community-based learning showed the most positive outcome, quality of studies assessing their effectiveness was only average as they mostly relied on small samples without control groups. In the same time, the effects of volunteering in student-run free clinics were not clear, although Smith et al. [58], who included the largest sample of volunteer medical students, reported that volunteering in student-run free clinics did improve MS-ATU. Moreover, studies evaluating effectiveness of educational interventions generally had small samples; thus, their results may be explained mostly by a lack of statistical power. We could also assume that using a questionnaire to capture the attitudes of students may not be as useful as evaluation based on experiential assessments such as Objective Structured Clinical Examinations (OSCEs). OSCES indeed offer an effective means to evaluate behavioral skills, empathetic posture or relational qualities in medical students toward the underserved [85], rather than only relying on self-reported attitudes through rating scales. Routinely measuring MS-ATU in medical schools using validated scales and/or OSCEs may be an effective mean to evaluate their effectiveness in training socially accountable medical students.

### Strengths and limitations

This is the first systematic review and meta-analysis evaluating the literature on change in attitudes toward the underserved throughout medical education. Our review includes a number of good-quality studies and a large sample of medical students. The results are convergent across studies and over time, giving a high level of confidence to our results. Our results stress the need for medical schools to improve their efforts to enhance commitment toward social responsibility. The findings are thus concordant with the call of Smitherman, Baker & Wilson [9] for medical schools to add a fourth mission – social responsibility – to the traditional tripartite mission of education, research, and clinical care.

Our systematic review had several limitations. First, over 90% of the studies reviewed were performed in high-income countries from the global-North. A recent study demonstrated that social attitudes in medical students are strongly influenced by societal social attitudes [86]. Moreover, idiosyncrasies of the medical systems in the high-income countries may also negatively influence the change in MS-ATU during medical education. Thus, the generalizability of our conclusions has to be questioned and more studies need to be conducted in other countries and in diverse medical education environments to better understand associations between MS-ATU and medical education. Second, we performed a meta-analysis considering only the factors associated with medical students’ attitudes, because statistical analyses were too heterogeneous for the change in MS-ATU and the effectiveness of educational interventions. Regardless, results were convergent in all studies. Third, studies had considerable heterogeneity in terms of outcomes and design. Use of several scales to assess MS-ATU is a serious limitation for generalizability of our results. Moreover, the assessment of MS-IWU did not rely on the use of any scale, which may also have increased the heterogeneity of outcomes and results. Fourth, we choose to exclude qualitative studies of the systematic review to limit the heterogeneity of data, which may have impeded the exhaustiveness of our results. Notably, qualitative studies could offer an in-depth capture of the attitudes and career choices of medical students. Fifth, we only included English-language articles and thus putatively induce a publication bias. Finally, medical students’ attitudes represent a limited part of the competencies related to social accountability. Other outcomes, such as knowledge and skills of medical students, should also be assessed in systematic reviews of the literature to fully understand the impact of medical schools on training socially accountable students.

## Conclusions

Despite increased interest among medical schools to inculcate social responsibility in their students, attitudes of medical students toward the underserved decline throughout medical education. This remains true even in more recent studies, after social responsibility programs have begun to be implemented. Educational interventions dedicated to improving attitudes of medical students show encouraging but mixed results. The generalizability of our results is limited by the great number of U.S. studies included in our systematic review. More evidence from European or low- and middle-income countries is needed to better understand associations between medical students’ attitudes and their education regarding health disparities.

## Supplementary Information

**Additional file 1.**

## Data Availability

Data supporting the findings of this study are available from the corresponding author (EL) on request at edouard.leaune@ch-le-vinatier.fr

## Abbreviations

MS-ATU: Medical students attitudes toward the underserved
MS-IWU: Medical students intention to work with the underserved

## References

[1] Scammon DL, Li LB, Williams SD (1995). Increasing the supply of providers for the medically underserved: marketing and public policy issues. J Public Policy Marketing.

[2] Marmot M (2005). Social determinants of health inequalities. Lancet..

[3] Hutchinson P, Morelli V (2017). International comparisons in underserved health: issues, policies, needs and projections. Prim Care.

[4] Lund C, Brooke-Sumner C, Baingana F (2018). Social determinants of mental disorders and the sustainable development goals: a systematic review of reviews. Lancet Psychiatry.

[5] Dickman SL, Himmelstein DU, Woolhandler S (2017). Inequality and the health-care system in the USA. Lancet..

[6] Anthony D, El Rayess F, Esquibel AY, George P, Taylor J (2014). Building a workforce of physicians to care for underserved patients. R I Med J.

[7] Reeve C, Woolley T, Ross SJ (2017). The impact of socially-accountable health professional education: a systematic review of the literature. Med Teach.

[8] Edelman A, Taylor J, Ovseiko PV, Topp SM (2018). The role of academic health centres in improving health equity: a systematic review. J Health Organ Manag.

[9] Smitherman HC, Baker RS, Wilson MR (2019). Socially accountable academic health centers: pursuing a quadripartite mission. Acad Med.

[10] Boelen C, Woollard B (2009). Social accountability and accreditation: a new frontier for educational institutions. Med Educ.

[11] Ventres W, Dharamsi S (2015). Socially accountable medical education-the REVOLUTIONS framework. Acad Med.

[12] Crandall SJ, Volk RJ, Loemker V (1993). Medical students' attitudes toward providing care for the underserved. Are we training socially responsible physicians?. JAMA..

[13] Crandall SJ, Volk RJ, Cacy D (1997). A longitudinal investigation of medical student attitudes toward the medically indigent. Teach Learn Med.

[14] Griffin B, Porfeli E, Hu W (2017). Who do you think you are? Medical student socioeconomic status and intention to work in underserved areas. Adv Health Sci Educ Theory Pract.

[15] Parlow J, Rothman A (1974). Attitudes towards social issues in medicine of five health science facilities. Soc Sci Med.

[16] Dornbush RL, Singer P, Brownstein EJ, Freedman AM (1985). Maintenance of psychosocial attitudes in medical students. Soc Sci Med.

[17] Yun SH, Weaver RD (2010). Development and validation of a short form of the attitude toward poverty scale. Adv Soc Work.

[18] Jilani D, Fernandes A, Borges N (2015). Pre-clinical versus clinical medical students’ attitudes towards the poor in the United States. J Educ Eval Health Prof.

[19] Eron LD (1955). Effect of medical education on medical students' attitudes. J Med Ed.

[20] Ko M, Edelstein RA, Heslin KC (2005). Impact of the University of California, los Angeles/Charles R. Drew University medical education program on medical students’ intentions to practice in underserved areas. Acad Med.

[21] Larkins S, Johnston K, Hogenbirk JC (2018). Practice intentions at entry to and exit from medical schools aspiring to social accountability: findings from the training for health equity network graduate outcome study. BMC Med Educ..

[22] Liberati A, Altman DG, Tetzlaff J (2009). The PRISMA statement for reporting systematic reviews and meta-analyses of studies that evaluate healthcare interventions: explanation and elaboration. BMJ..

[23] Oxford Centre for Evidence-Based Medicine. Levels of evidence : https://www.cebm.net/2009/06/oxford-centre-evidence-based-medicine-levels-evidence-march-2009/. Accessed January 24, 2019.

[24] Cook DA, Reed DA (2015). Appraising the quality of medical education research methods: the medical education research study quality instrument and the Newcastle-Ottawa scale-education. Acad Med.

[25] Cohen J (1988). Statistical power analysis for the behavioral sciences.

[26] Borenstein M, Hedges L, Higgins J, Rothstein H (2005). Comprehensive meta-analysis version 2.

[27] Higgins JPT, Thomas J, Chandler J, Cumpston M, Li T, Page MJ, Welch VA (2019). Cochrane Handbook for Systematic Reviews of Interventions version 6.0. Cochrane.

[28] Cooper H, Hedges LV, Valentine JC (2009). The handbook of research synthesis and meta-analysis. Russell Sage Foundation; 2009.

[29] Higgins JP, Thompson SG, Deeks JJ, Altman DG (2003). Measuring inconsistency in meta-analyses. BMJ.

[30] Egger M, Davey Smith G, Schneider M, Minder C (1997). Bias in meta-analysis detected by a simple, graphical test. BMJ.

[31] Maisiak R, Meredith RL, Boker J, Rider-Gordon B, Scott LK (1980). Change in medical students’ attitudes toward social issues after a course in behavioral science. J Psych Educ.

[32] Ewan CE (1987). Attitudes to social issues in medicine: a comparison of first-year medical students with first-year students in non-medical faculties. Med Educ.

[33] Ewan CE (1988). Social issues in medicine: a follow-up comparison of senior-year medical students’ attitudes with contemporaries in non-medical faculties. Med Educ.

[34] Dornbush RL, Richman S, Singer P, Brownstein EJ (1991). Medical school, psychosocial attitudes, and gender. J Am Med Womens Assoc.

[35] Tippets EA, Westpheling KM (1993). Practice in medically underserved areas: medical studentsʼ attitudes and intents. Acad Medicine.

[36] Campos-Outcalt D, Chang S, Pust R, Johnson L (1997). Commitment to the underserved: evaluating the effect of an extracurricular medical student program on career choice. Teach Learn Med.

[37] O’Toole TP, Hanusa BH, Gibbon JL, Boyles SH (1999). Experiences and attitudes of residents and students influence voluntary service with homeless populations. J Gen Intern Med.

[38] Weitzman CC, Freudigman K, Schonfeld DJ, Leventhal JM (2000). Care to underserved children: residents’ attitudes and experiences. Pediatrics..

[39] Markham FW, Sawhney HK, Butler JA, Diamond JJ (2001). The changing perceptions of junior medical students about the current U.S. health care system after a seminar series. J Community Health.

[40] Weissman JS, Campbell EG, Gokhale M, Blumenthal D (2001). Residents’ preferences and preparation for caring for underserved populations. J Urban Health.

[41] Godkin M, Savageau J (2003). The effect of medical students’ international experiences on attitudes toward serving underserved multicultural populations. Fam Med.

[42] Tavernier LA, Connor PD, Gates D, Wan JY (2003). Does exposure to medically underserved areas during training influence eventual choice of practice location?. Med Educ.

[43] Schwartz PL, Loten EG (2004). Effect of year in school on medical students’ perceptions evaluated with the cognitive behavior survey, attitudes toward social issues in medicine survey, and learning environment questionnaire. Teach Learn Med.

[44] Wilson E, Grumbach K, Huebner J, Agrawal J, Bindman AB (2004). Medical student, physician, and public perceptions of health care disparities. Fam Med.

[45] Woloschuk W, Harasym PH, Temple W (2004). Attitude change during medical school: a cohort study. Med Educ.

[46] Cox E, Koscik R, Olson C (2006). Caring for the underserved blending service learning and a web-based curriculum. Am J Prev Med.

[47] Godkin MA, Savageau JA, Fletcher KE (2006). Effect of a global longitudinal pathway on medical students’ attitudes toward the medically indigent. Teach Learn Med.

[48] Buchanan D, Rohr L, Stevak L, Sai T (2007). Documenting attitude changes towards homeless people: comparing two standardised surveys. Med Educ.

[49] Crandall SJ, Reboussin BA, Michielutte R, Anthony JE, Naughton MJ (2007). Medical students’ attitudes toward underserved patients: a longitudinal comparison of problem-based and traditional medical curricula. Adv Health Sci Educ Theory Pract.

[50] Ko M, Heslin KC, Edelstein RA, Grumbach K (2007). The role of medical education in reducing health care disparities: the first ten years of the UCLA/drew medical education program. J Gen Intern Med.

[51] Crandall SJ, Davis SW, Broeseker AE, Hildebrandt C (2008). A longitudinal comparison of pharmacy and medical students’ attitudes toward the medically underserved. Am J Pharm Educ.

[52] Dyrbye LN, Massie FS, Eacker A (2010). Relationship between burnout and professional conduct and attitudes among US medical students. JAMA..

[53] Huang WY, Malinow A (2010). Curriculum and evaluation results of a third-year medical student longitudinal pathway on underserved care. Teach Learn Med.

[54] Wayne S, Dellmore D, Serna L, Jerabek R, Timm C, Kalishman S (2011). The association between intolerance of ambiguity and decline in medical students’ attitudes toward the underserved. Acad Med.

[55] Sheu L, Lai CJ, Coelho AD (2012). Impact of student-run clinics on preclinical sociocultural and interprofessional attitudes: a prospective cohort analysis. J Health Care Poor Underserved.

[56] Boscardin CK, Grbic D, Grumbach K, O’Sullivan P (2014). Educational and individual factors associated with positive change in and reaffirmation of medical students’ intention to practice in underserved areas. Acad Med.

[57] Caulfield M, Andolsek K, Grbic D, Roskovensky L (2014). Ambiguity tolerance of students matriculating to U.S. medical schools. Acad Med.

[58] Smith SD, Yoon R, Johnson ML, Natarajan L, Beck E (2014). The effect of involvement in a student-run free clinic project on attitudes toward the underserved and interest in primary care. J Health Care Poor Underserved.

[59] Borracci RA, Arribalzaga EB, Couto JL (2015). Factors affecting willingness to practice medicine in underserved areas: a survey of argentine medical students. Rural Remote Health.

[60] Cox ED, Koscik RL, Behrmann AT (2015). Long term outcomes of a curriculum on care for the underserved. J Natl Med Assoc.

[61] Girotti JA, Loy GL, Michel JL, Henderson VA (2015). The urban medicine program: developing physician-leaders to serve underserved urban communities. Acad Med.

[62] Larkins S, Michielsen K, Iputo J (2015). Impact of selection strategies on representation of underserved populations and intention to practise: international findings. Med Educ.

[63] Stephens MB, Landers G, Davis SW, Durning SJ, Crandall SJ (2015). Medical student attitudes toward the medically underserved: the USU perspective. Mil Med.

[64] Laraque-Arena D, Frintner MP, Cull WL (2016). Underserved areas and pediatric resident characteristics: is there reason for optimism?. Acad Pediatr.

[65] Leung LB, Simmons JE, Ho J, Anselin E, Yalamanchili R, Rabatin JS (2016). A five-year evolution of a student-led elective on health disparities at the Alpert medical school. R I Med J.

[66] Gatell VI, Nguyen T, Anderson EE, McCarthy MP, Hardt JJ (2017). Characteristics of medical students planning to work in medically underserved settings. J Health Care Poor Underserved.

[67] O’Connell TF, Ham SA, Hart TG, Curlin FA, Yoon JD (2018). A national longitudinal survey of medical students’ intentions to practice among the underserved. Acad Med.

[68] Puddey IB, Playford DE, Mercer A (2017). Impact of medical student origins on the likelihood of ultimately practicing in areas of low vs high socio-economic status. BMC Med Educ..

[69] Tran K, Kovalskiy A, Desai A, Imran A, Ismail R, Hernandez C (2017). The effect of volunteering at a student-run free healthcare clinic on medical students’ self-efficacy, comfortableness, attitude, and interest in working with the underserved population and interest in primary care. Cureus.

[70] Briggs AM, Wang SY, Bhowmik S, Wasag J, Pinto-Powell RC (2018). The beyond the books program: improving medical student attitudes toward the underserved. Health Equity.

[71] Garcia AN, Kuo T, Arangua L, Pérez-Stable EJ (2018). Factors associated with medical school graduates’ intention to work with underserved populations: policy implications for advancing workforce diversity. Acad Med.

[72] Kost A, Evans D, Dobie S, Sanders E (2018). What is the impact of the underserved pathway program on graduates entering an underserved family medicine residency? Five-year findings from the University of Washington School of medicine. Acad Med.

[73] Woolley T, Cristobal F, Siega-Sur J (2018). Positive implications from socially accountable, community-engaged medical education across two Philippines regions. Rural Remote Health.

[74] Burkhardt J, DesJardins S, Gruppen L (2019). Diversity in emergency medicine: are we supporting a career interest in emergency medicine for everyone?. Ann Emergency Med.

[75] Godfrey S, Nickerson K, Amiel J, Lebwohl B (2019). Development of an online public health curriculum for medical students: the public health commute. BMC Med Educ.

[76] Heller M, Thomas AM, Peters SM, Düsterwald KM, Klausner JD (2019). An evaluation of patient and student experience at a longstanding student-run free clinic in Cape Town, South Africa. Cureus.

[77] Jacobs C, Seehaver A, Skiold-Hanlin S (2019). A longitudinal underserved community curriculum for family medicine residents. Fam Med.

[78] Phelan SM, Burke SE, Cunningham BA, Harden K, van Ryn M (2019). The effects of racism in medical education on students' decisions to practice in underserved or minority communities. Acad Med.

[79] Hafferty FW (1998). Beyond curriculum reform: confronting medicine’s hidden curriculum. Acad Med.

[80] Lawrence C, Mhlaba T, Stewart KA, Moletsane R, Gaede B, Moshabela M (2018). The hidden curricula of medical education: a scoping review. Acad Med.

[81] Neumann M, Edelhäuser F, Tauschel D (2011). Empathy decline and its reasons : a systematic review of studies with medical students and residents. Acad Med.

[82] Keis O, Schneider A, Heindl F, Huber-Lang M, Öchsner W, Grab-Kroll C (2019). How do German medical students perceive role models during clinical placements (“Famulatur”)? An empirical study. BMC Med Educ.

[83] Marrast LM, Zallman L, Woolhandler S, Bor DH, McCormick D (2014). Minority physicians' role in the care of underserved patients: diversifying the physician workforce may be key in addressing health disparities. JAMA Intern Med.

[84] Doobay-Persaud A, Adler MD, Bartell TR (2019). Teaching the social determinants of health in undergraduate medical education: a scoping review. J Gen Intern Med.

[85] Seifert LB, Schaack D, Jennewein L, Steffen B, Schulze J, Gerlach F, Sader R (2016). Peer-assisted learning in a student-run free clinic project increases clinical competence. Med Teach.

[86] Stefanovics EA, Rosenheck RA, He H, Ofori-Atta A, Cavalcanti M, Chiles C (2016). Medical student beliefs and attitudes toward mental illness across five nations. J Nerv Ment Dis.

