# Supplementary material for: Medical students attitudes toward and intention to work with the underserved: a systematic review and meta-analysis
Source: BMC Med Educ. 2021 Feb 24;21:129. doi: 10.1186/s12909-021-02517-x (PMC7905612; doi:10.1186/s12909-021-02517-x)
Supplement: Supplementary file 1 — Additional file 1. [file 12909_2021_2517_MOESM1_ESM.docx]

**Appendix. Algorithm used for the systematic electronic research**

(("education, medical"[MeSH Terms] OR ("education"[All Fields] AND "medical"[All Fields]) OR "medical education"[All Fields] OR ("medical"[All Fields] AND "education"[All Fields])) OR (medical student[All Fields] OR medical student/clinical[All Fields] OR medical student/community[All Fields] OR medical student/faculty[All Fields] OR medical student/health[All Fields] OR medical student/patient[All Fields] OR medical student/research[All Fields] OR medical student/residency[All Fields] OR medical student/surgical[All Fields] OR medical student/universidade[All Fields] OR medical student's[All Fields] OR medical student1s[All Fields] OR medical studentb[All Fields] OR medical studente[All Fields] OR medical studentitis[All Fields] OR medical students[All Fields] OR medical students/clinical[All Fields] OR medical students/education[All Fields] OR medical students/future[All Fields] OR medical students/residents[All Fields] OR medical students/school[All Fields] OR medical students/surgical[All Fields] OR medical students'[All Fields] OR medical students'association[All Fields] OR medical students'associations[All Fields] OR medical students'experiences[All Fields] OR medical students'knowledge[All Fields] OR medical students'performance[All Fields] OR medical students'research[All Fields] OR medical students's[All Fields] OR medical students'society[All Fields] OR medical students38[All Fields] OR medical students`attitudes[All Fields] OR medical students`exposure[All Fields] OR medical students`health[All Fields] OR medical students`perception[All Fields] OR medical students`perceptions[All Fields] OR medical studentsa[All Fields] OR medical studentsdoctors[All Fields] OR medical studentselizabeth[All Fields] OR medical studentship[All Fields] OR medical studentships[All Fields] OR medical studentsmentors[All Fields] OR medical studentsn[All Fields] OR medical studentsself[All Fields] OR medical studentssix[All Fields]) OR (medical trainee[All Fields] OR medical trainee's[All Fields] OR medical trainees[All Fields] OR medical trainees'[All Fields] OR medical traineeship[All Fields] OR medical traineeships[All Fields]) OR (medical[All Fields] AND interns[All Fields]) AND "OR "[All Fields] AND (medical resident[All Fields] OR medical resident's[All Fields] OR medical residential[All Fields] OR medical residentnurse[All Fields] OR medical residents[All Fields] OR medical residents'[All Fields] OR medical residentsa[All Fields])) AND (("attitude"[MeSH Terms] OR "attitude"[All Fields] OR "attitudes"[All Fields]) OR ("education"[Subheading] OR "education"[All Fields] OR "training"[All Fields] OR "education"[MeSH Terms] OR "training"[All Fields]) OR program[All Fields] OR trend[All Fields]) AND (underserved[All Fields] OR social[All Fields] OR ("poverty"[MeSH Terms] OR "poverty"[All Fields] OR "poor"[All Fields]))
